# 1-Ethyl-2-phenyl-3-[2-(tri­methyl­sil­yl)ethyn­yl]-1*H*-indole

**DOI:** 10.1107/S1600536813012671

**Published:** 2013-05-18

**Authors:** Iaroslav Baglai, Valérie Maraval, Carine Duhayon, Remi Chauvin

**Affiliations:** a Department of Chemistry, Taras Shevchenko National University of Kyiv, Volodymyrska 64, 01033 Kyiv, Ukraine; b CNRS, LCC (Laboratoire de Chimie de Coordination), 205, route de Narbonne, F-31077 Toulouse, France; c Universite de Toulouse, UPS, INPT, LCC, F-31077 Toulouse, France

## Abstract

The title compound, C_21_H_23_NSi, was synthesized by Sonogashira-type reaction of 1-ethyl-3-iodo-2-phenyl-1*H*-indole with tri­methyl­silyl­acetyl­ene. The indole ring system is nearly planar [maximum atomic deviation = 0.0244 (15) Å] and is oriented at a dihedral angle of 51.48 (4)° with respect to the phenyl ring. The supramolecular aggregation is completed by weak C—H⋯π inter­actions of the methylene and phenyl groups with the benzene and pyrrole rings of the indole ring system. The methyl groups of the tri­methyl­silyl unit are equally disordered over two sets of sites.

## Related literature

For background to indoles, see: Huang *et al.* (2004 ▶); Seferoğlu *et al.* (2007*a* ▶,*b* ▶). For the synthesis and properties of indoles, see: Ruiz *et al.* (2012 ▶); Shiri (2012 ▶); Hussain *et al.* (2011 ▶); Prateeptongkum *et al.* (2010 ▶); Rives *et al.* (2012 ▶).
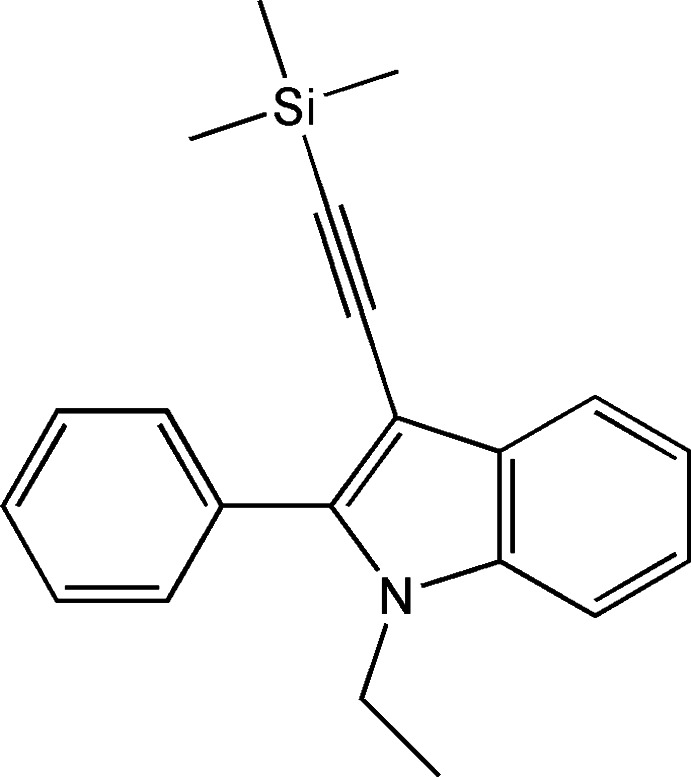


## Experimental

### 

#### Crystal data

C_21_H_23_NSi
*M* *_r_* = 317.51
Monoclinic, 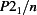

*a* = 12.6271 (6) Å
*b* = 9.3928 (5) Å
*c* = 16.6616 (8) Å
β = 111.954 (2)°
*V* = 1832.83 (16) Å^3^
*Z* = 4
Mo *K*α radiation
μ = 0.13 mm^−1^
*T* = 100 K
0.20 × 0.20 × 0.06 mm

#### Data collection

Bruker Kappa APEXII diffractometer
Absorption correction: multi-scan (*SADABS*; Bruker, 2001 ▶) *T*_min_ = 0.90, *T*_max_ = 0.99
26426 measured reflections
4320 independent reflections
3254 reflections with *I* > 2.0σ(*I*)
*R*_int_ = 0.032

#### Refinement

*R*[*F* ^2^ > 2σ(*F* ^2^)] = 0.038
*wR*(*F* ^2^) = 0.039
*S* = 1.12
3032 reflections
235 parameters
6 restraints
H-atom parameters constrained
Δρ_max_ = 0.31 e Å^−3^
Δρ_min_ = −0.30 e Å^−3^

### 

Data collection: *APEX2* (Bruker, 2006 ▶); cell refinement: *SAINT* (Bruker, 2006 ▶); data reduction: *SAINT*; program(s) used to solve structure: *SHELXS97* (Sheldrick, 2008 ▶); program(s) used to refine structure: *CRYSTALS* (Betteridge *et al.*, 2003 ▶); molecular graphics: *CAMERON* (Watkin *et al.*, 1996 ▶); software used to prepare material for publication: *CRYSTALS*.

## Supplementary Material

Crystal structure: contains datablock(s) global, I. DOI: 10.1107/S1600536813012671/xu5700sup1.cif

Structure factors: contains datablock(s) I. DOI: 10.1107/S1600536813012671/xu5700Isup2.hkl

Supplementary material file. DOI: 10.1107/S1600536813012671/xu5700Isup3.cdx

Supplementary material file. DOI: 10.1107/S1600536813012671/xu5700Isup4.cml

Additional supplementary materials:  crystallographic information; 3D view; checkCIF report

## Figures and Tables

**Table 1:** Hydrogen-bond geometry (Å, °) *Cg*4 and *Cg*5 are the centroids of the pyrrole and benzene rings, respectively, of indole ring system.

*D*—H⋯*A*	*D*—H	H⋯*A*	*D*⋯*A*	*D*—H⋯*A*
C10—H102⋯*Cg*5^i^	0.98	2.66	3.3512 (18)	128
C16—H161⋯*Cg*5^ii^	0.95	2.79	3.5122 (17)	133
C17—H171⋯*Cg*4^ii^	0.95	2.81	3.4515 (17)	125

Symmetry codes: (i) 
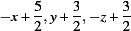
; (ii) 
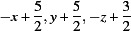
.

**Fig. 1.**
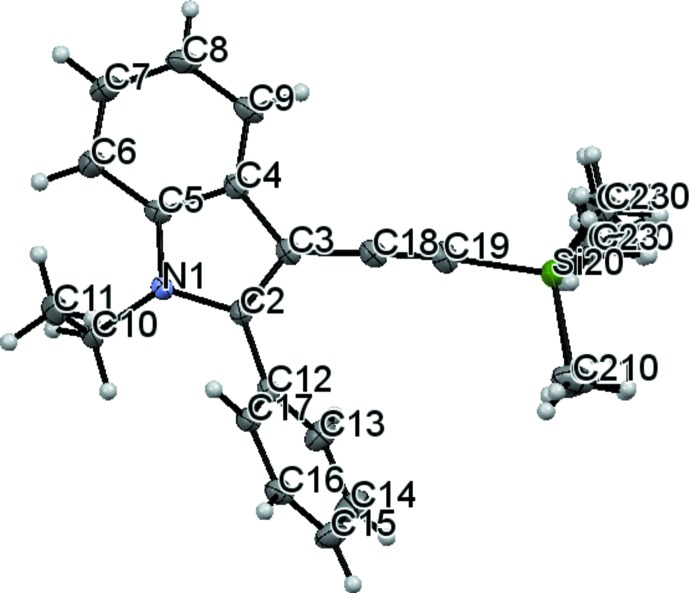
The molecular structure of the title compound, showing the atomic numbering scheme. Displacement ellipsoids are drawn at the 50% probability level.

**Fig. 2.**
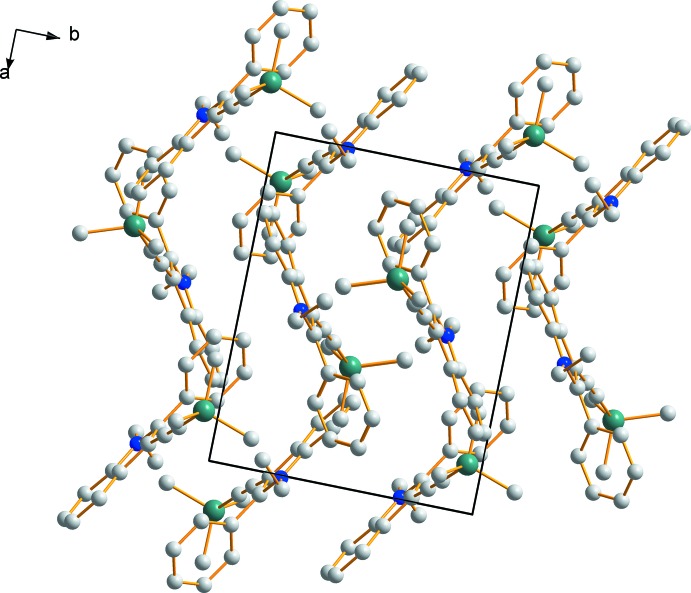
Packing diagram of the title compound, viewed along the c axis.

**Fig. 3.**
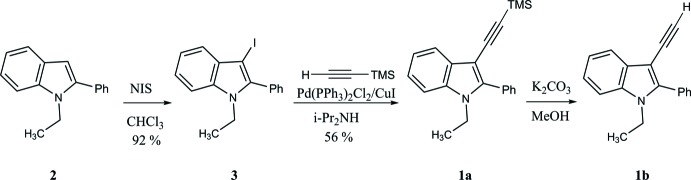
The reaction scheme.

## supplementary crystallographic information

### Comment

Indole is one of the three heterocycles occurring in the 20 standard natural amino-acids (in (*L*)-trhyptophane). Its unique bicyclic aromatic structure makes it a key rigid structural basis of one of the largest classes of alkaloids, comprising more than 4000 natural products (Huang *et al.*, 2004; Seferoğlu *et al.*, 2007*a*,*b*; Ruiz *et al.*, 2012). The synthesis and functionalization of indoles have been the object of research for over one and a half century (Shiri, 2012). Few examples of Sonogashira-type reactions on the indole core have been described (Hussain *et al.*, 2011; Prateeptongkum *et al.*, 2010). In view of obtaining 1-ethyl-3-ethynyl-2-phenyl-1*H*-indole 1 b as key building block for the synthesis of highly π-frustrated *carbo*-benzenic chromophores (Rives *et al.* 2012), the title compound (1a) was synthesized (Fig. 3), and we herein report on its crystal structure.

In the molecule (Fig. 1), the indole ring is almost planar [maximum deviation for ring atoms = 0.0244 (15) Å], and the dihedral angle between the pyrrole and benzene ring of the indole system is equal to 1.83 (5)°. Methyl groups of the trimethylsilyl moieties are disordered into two positions with the corresponding occupancy of 0.5 for each part. The whole molecule is almost planar excluding methyl groups of trimethylsilyl and ethyl moieties as well as the benzene ring [maximum deviation for atoms = 0.0539 (16) Å]. The interplanar angle between indole and phenyl planes is equal to 51.48 (4)°. Inspite of the three aromatic rings of the molecule, the packing shown in Fig. 2 does not reveal any particular intermolecular π-stacking or columnar arrangement but weak C—H···π interactions (Table 1).

### Experimental

The title compound 1a was prepared by the following two-step procedure (see Fig. 3) from commercially available 1-ethyl-2-phenyl-1*H*-indole 2*via* 1-ethyl-3-iodo-2-phenyl-1*H*-indole 3.

1-ethyl-3-iodo-2-phenyl-1*H*-indole (3).

To a solution of 1-ethyl-2-phenyl-1*H*-indole 2 (0.5 g, 2.26 mmol) in CHCl_3_ (30 ml) at 0 °C was added *N*-iodosuccinimide (0.535 g, 2.37 mmol) as a small portions over 5 min. The mixture was then stirred at the same temperature for 3 h. After evaporating the solvent, the residue was extracted in dichloromethane and washed with H_2_O. The organic layer was separated and dried over MgSO_4_. The solvent was removed under reduced pressure, and the product was purified by silica gel chromatography using a mixture of acetone and pentane (2:98) as an eluent (yield 92%, 0.72 g). *R*_f_= 0.26.

M. p. 93 °C. ^1^H NMR (CDCl_3_) *δ* 7.59 - 7.44 (m, 6 H, *H*9 Ind, *o-, m-, p-*Ph), 7.39 - 7.22 (m, 3 H, *H*6, *H*7, *H*8 Ind), 4.16 (q, *J* = 7.1 Hz, 2 H, CH_2_), 1.26 (t, *J* = 7.2 Hz, 3 H, CH_3_). ^13^C NMR (101 MHz, CDCl_3_) *δ* 141.4 (*C*2 Ind), 136.5 (*C*5 Ind), 132.0 (*i*-Ph), 130.8 (*m-*Ph), 130.6 (*C*4 Ind), 128.9 (*p*-Ph), 128.5 (*o*-Ph), 122.8, 121.6, 120.6, (*C*7, *C*8, *C*9 Ind), 110.0 (*C*6 Ind), 59.5 (*C*3 Ind), 39.9 (CH_2_), 15.5 (CH_3_). HRMS (DCI/CH_4_): *m/z* calcd for C_16_H_14_NI: 347.0171, found: 347.0165.

1-ethyl-2-phenyl-3-[2-(trimethylsilyl)ethynyl]-1*H*-indole (1a).

CuI (42 mg, 0.21 mmol) and Pd(PPh_3_)_2_Cl_2_ (60 mg, 0.09 mmol) were added to 1-ethyl-3-iodo-2-phenyl-1*H*-indole 3 (1.04 g, 3.0 mmol) under argon atmosphere. Then, freshly distilled diisopropylamine (15 ml) was added, and the mixture was stirred for 20 min, Me_3_SiC≡CH (1.0 ml, 6.52 mmol) was added to the mixture and the suspension was stirred for 60 h at room temperature before adding Et_2_O (20 ml). The mixture was filtrated through Celite and the filtrate was evaporated, the residue was re-dissolved in Et_2_O and washed with 10% HCl, water and NaHCO_3_. The resulting organic solution was dried over anhydrous MgSO_4_, the solvent was removed under reduced pressure, and the brown residue was purified by silica gel chromatography using a mixture of ether and pentane (1:99) as an eluent (yield 56%, 0.53 g). *R*_f_(99/5 = C_5_H_12_/Ether) = 0.47. The title compound was dissolved in Et_2_O and CH_2_Cl_2_ (1:1 mixture). After slow evaporation over two days, crystals of 1a suitable for X-ray diffraction analysis were obtained.

M. p. 86 °C. ^1^H NMR (400 MHz, CDCl_3_) *δ* 7.92 (d, *J* = 7.4 Hz, 1 H, *H*9 Ind), 7.72 (d, *J* = 7.9 Hz, 2 H, *o-*Ph), 7.61- 7.33 (m, 6 H, *H*6, *H*7, *H*8 Ind, *m-,p-*Ph), 4.26 (q, *J* = 7.1 Hz, 2 H, CH_2_), 1.38 (t, *J* = 7.1 Hz, 3 H, CH_3_), 0.34. (s, 9 H, TMS). ^13^C NMR (101 MHz, CDCl_3_) *δ* 144.3 (*C*2 Ind), 135.9 (*C*5 Ind), 131.1 (*i*-Ph), 130.2 (*m*-Ph), 129.4 (*C*4 Ind), 128.6 (*p*-Ph), 128.4 (*o*-Ph), 122.9, 120.9, 120.3 (*C*7, *C*8, *C*9 Ind), 110.2 (*C*6 Ind), 100.0, 97.4, 96.4 (*C*3 Ind, –C≡C–), 39.3 (CH_2_), 15.3 (CH_3_), 0.43 (Si(CH_3_)_3_). HRMS (DCI/CH_4_): *m/z* calcd for C_21_H_23_NSi: 317.1600, found: 317.1614.

### Refinement

The H atoms were located in a difference Fourier map and refined with riding constraints. Methyl groups are disordered over two positions in site occupancy ratio of 0.5:0.5.

### Crystal data

**Table:**

C_21_H_23_NSi	*F*(000) = 680
*M**_r_* = 317.51	*D*_x_ = 1.151 Mg m^−^^3^
Monoclinic, *P*2_1_/*n*	Melting point: 359 K
Hall symbol: -P 2yn	Mo *K*α radiation, λ = 0.71073 Å
*a* = 12.6271 (6) Å	Cell parameters from 9299 reflections
*b* = 9.3928 (5) Å	θ = 3–28°
*c* = 16.6616 (8) Å	µ = 0.13 mm^−^^1^
β = 111.954 (2)°	*T* = 100 K
*V* = 1832.83 (16) Å^3^	Planar, colourless
*Z* = 4	0.20 × 0.20 × 0.06 mm

### Data collection

**Table:**

Bruker Kappa APEXII diffractometer	3254 reflections with *I* > 2.0σ(*I*)
Graphite monochromator	*R*_int_ = 0.032
φ and ω scans	θ_max_ = 28.1°, θ_min_ = 2.5°
Absorption correction: multi-scan (*SADABS*; Bruker, 2001)	*h* = −16→16
*T*_min_ = 0.90, *T*_max_ = 0.99	*k* = −12→12
26426 measured reflections	*l* = −22→21
4320 independent reflections	

### Refinement

**Table:**

Refinement on *F*	Primary atom site location: structure-invariant direct methods
Least-squares matrix: full	Hydrogen site location: difference Fourier map
*R*[*F*^2^ > 2σ(*F*^2^)] = 0.038	H-atom parameters constrained
*wR*(*F*^2^) = 0.039	[*w*eight] = 1.0/[A_0_*T_0_(x) + A_1_*T_1_(x) ··· + A_n-1_]*T_n-1_(x)] where A_i_ are 0.191, 0.134 and 0.621E-01, and x = *F* /*F*max
*S* = 1.12	(Δ/σ)_max_ = 0.001
3032 reflections	Δρ_max_ = 0.31 e Å^−^^3^
235 parameters	Δρ_min_ = −0.30 e Å^−^^3^
6 restraints	

### Special details

**Table:**

Refinement. Structure was refined by full-matrix least-squares procedures on F using the programs of the PC version of CRYSTALS, with 3032 reflexions [I>3.0σ(I)].

### Fractional atomic coordinates and isotropic or equivalent isotropic displacement parameters (Å^2^)

**Table:**

	*x*	*y*	*z*	*U*_iso_*/*U*_eq_	Occ. (<1)
N1	0.99268 (10)	0.72549 (13)	0.49251 (8)	0.0196	
C2	0.93192 (11)	0.79040 (15)	0.53618 (9)	0.0184	
C3	0.98715 (12)	0.76670 (15)	0.62446 (9)	0.0195	
C4	1.08714 (12)	0.68318 (16)	0.63574 (9)	0.0207	
C5	1.08685 (11)	0.65774 (16)	0.55223 (9)	0.0211	
C6	1.17008 (13)	0.57357 (17)	0.53885 (10)	0.0267	
C7	1.25572 (13)	0.51899 (18)	0.61168 (11)	0.0299	
C8	1.25924 (13)	0.54708 (17)	0.69552 (10)	0.0284	
C9	1.17547 (13)	0.62798 (16)	0.70832 (10)	0.0243	
C10	0.96037 (13)	0.71346 (17)	0.39867 (9)	0.0241	
C11	1.03390 (14)	0.80491 (19)	0.36501 (10)	0.0309	
C12	0.82652 (11)	0.87255 (15)	0.49367 (9)	0.0183	
C13	0.73271 (13)	0.84517 (17)	0.51658 (11)	0.0261	
C14	0.63450 (13)	0.92593 (18)	0.48225 (12)	0.0315	
C15	0.62723 (13)	1.03523 (16)	0.42414 (11)	0.0278	
C16	0.71982 (12)	1.06377 (16)	0.40118 (10)	0.0236	
C17	0.81897 (12)	0.98331 (16)	0.43583 (9)	0.0206	
C18	0.95193 (12)	0.81975 (15)	0.69075 (9)	0.0209	
C19	0.91972 (13)	0.86562 (16)	0.74527 (9)	0.0235	
Si20	0.85785 (3)	0.94562 (4)	0.81844 (3)	0.0218	
C23	0.9113 (3)	1.1387 (3)	0.8372 (2)	0.0240	0.5000
C230	0.9514 (3)	1.0854 (4)	0.8847 (2)	0.0301	0.5000
C220	0.8439 (3)	0.7986 (4)	0.8949 (2)	0.0293	0.5000
C22	0.9054 (3)	0.8510 (4)	0.9200 (2)	0.0302	0.5000
C210	0.7149 (4)	1.0146 (5)	0.7538 (3)	0.0464	0.5000
C21	0.7003 (3)	0.9480 (5)	0.7620 (3)	0.0345	0.5000
H61	1.1681	0.5558	0.4825	0.0324*	
H71	1.3139	0.4604	0.6047	0.0374*	
H81	1.3203	0.5084	0.7444	0.0353*	
H91	1.1767	0.6449	0.7647	0.0307*	
H102	0.9687	0.6133	0.3855	0.0301*	
H101	0.8813	0.7425	0.3710	0.0299*	
H111	1.0099	0.7914	0.3042	0.0467*	
H112	1.1132	0.7787	0.3925	0.0468*	
H113	1.0230	0.9044	0.3756	0.0470*	
H131	0.7377	0.7680	0.5565	0.0325*	
H141	0.5703	0.9070	0.4976	0.0385*	
H151	0.5591	1.0887	0.4017	0.0332*	
H161	0.7156	1.1379	0.3613	0.0296*	
H171	0.8831	1.0045	0.4207	0.0257*	
H211	0.6822	0.9988	0.7107	0.0406*	0.5000
H212	0.6671	0.9920	0.7969	0.0406*	0.5000
H213	0.6727	0.8555	0.7497	0.0406*	0.5000
H2101	0.6698	0.9412	0.7211	0.0595*	0.5000
H2102	0.6816	1.0516	0.7905	0.0595*	0.5000
H2103	0.7212	1.0859	0.7171	0.0595*	0.5000
H231	0.8869	1.1873	0.7848	0.0306*	0.5000
H232	0.9907	1.1381	0.8613	0.0306*	0.5000
H233	0.8833	1.1838	0.8749	0.0306*	0.5000
H2301	0.9584	1.1579	0.8489	0.0395*	0.5000
H2302	1.0230	1.0473	0.9158	0.0395*	0.5000
H2303	0.9201	1.1220	0.9228	0.0395*	0.5000
H221	0.8782	0.7581	0.9102	0.0387*	0.5000
H222	0.9848	0.8500	0.9442	0.0387*	0.5000
H223	0.8773	0.8957	0.9578	0.0387*	0.5000
H2201	0.7966	0.7265	0.8625	0.0367*	0.5000
H2202	0.9160	0.7618	0.9260	0.0367*	0.5000
H2203	0.8131	0.8365	0.9330	0.0367*	0.5000

### Atomic displacement parameters (Å^2^)

**Table:**

	*U*^11^	*U*^22^	*U*^33^	*U*^12^	*U*^13^	*U*^23^
N1	0.0187 (5)	0.0257 (6)	0.0131 (6)	0.0054 (5)	0.0045 (4)	−0.0013 (5)
C2	0.0192 (6)	0.0204 (7)	0.0163 (7)	−0.0005 (5)	0.0073 (5)	−0.0032 (5)
C3	0.0211 (6)	0.0193 (7)	0.0174 (7)	−0.0013 (5)	0.0064 (5)	−0.0019 (5)
C4	0.0214 (7)	0.0211 (7)	0.0180 (7)	−0.0004 (5)	0.0054 (5)	−0.0022 (5)
C5	0.0186 (6)	0.0257 (7)	0.0161 (7)	0.0021 (6)	0.0031 (5)	−0.0008 (6)
C6	0.0232 (7)	0.0334 (9)	0.0225 (8)	0.0066 (6)	0.0074 (6)	−0.0021 (6)
C7	0.0214 (7)	0.0335 (9)	0.0317 (9)	0.0081 (6)	0.0063 (6)	−0.0017 (7)
C8	0.0212 (7)	0.0299 (8)	0.0247 (8)	0.0040 (6)	−0.0022 (6)	0.0017 (7)
C9	0.0258 (7)	0.0250 (7)	0.0160 (7)	−0.0006 (6)	0.0007 (5)	−0.0006 (6)
C10	0.0253 (7)	0.0314 (8)	0.0133 (7)	0.0077 (6)	0.0044 (6)	−0.0040 (6)
C11	0.0327 (8)	0.0436 (10)	0.0181 (8)	0.0098 (7)	0.0114 (6)	0.0030 (7)
C12	0.0172 (6)	0.0197 (6)	0.0164 (6)	0.0002 (5)	0.0045 (5)	−0.0045 (5)
C13	0.0244 (7)	0.0233 (7)	0.0341 (8)	0.0027 (6)	0.0147 (6)	0.0031 (6)
C14	0.0220 (7)	0.0311 (9)	0.0460 (10)	0.0015 (6)	0.0179 (7)	0.0030 (7)
C15	0.0198 (7)	0.0257 (8)	0.0353 (9)	0.0051 (6)	0.0073 (6)	−0.0019 (6)
C16	0.0228 (7)	0.0241 (7)	0.0199 (7)	−0.0001 (6)	0.0034 (5)	−0.0004 (6)
C17	0.0171 (6)	0.0275 (7)	0.0161 (7)	−0.0011 (5)	0.0049 (5)	−0.0018 (6)
C18	0.0239 (7)	0.0195 (7)	0.0179 (7)	−0.0017 (5)	0.0061 (6)	−0.0004 (5)
C19	0.0300 (7)	0.0220 (7)	0.0194 (7)	−0.0014 (6)	0.0102 (6)	−0.0011 (6)
Si20	0.02245 (19)	0.0247 (2)	0.0181 (2)	0.00257 (17)	0.00724 (15)	−0.00369 (17)
C23	0.0307 (15)	0.0225 (15)	0.0230 (16)	−0.0008 (13)	0.0148 (13)	−0.0054 (13)
C230	0.0341 (17)	0.0319 (18)	0.0258 (18)	−0.0038 (14)	0.0130 (14)	−0.0090 (15)
C220	0.0286 (16)	0.0391 (19)	0.0237 (17)	−0.0033 (15)	0.0139 (14)	−0.0031 (14)
C22	0.0373 (18)	0.0333 (18)	0.0224 (17)	−0.0011 (16)	0.0139 (15)	0.0016 (14)
C210	0.037 (2)	0.031 (2)	0.050 (3)	0.009 (2)	−0.0083 (19)	−0.012 (2)
C21	0.0283 (18)	0.036 (2)	0.039 (2)	−0.001 (2)	0.0127 (15)	0.000 (2)

### Geometric parameters (Å, º)

**Table:**

N1—C2	1.3811 (17)	C15—H151	0.945
N1—C5	1.3872 (18)	C16—C17	1.390 (2)
N1—C10	1.4653 (18)	C16—H161	0.951
C2—C3	1.3901 (19)	C17—H171	0.954
C2—C12	1.4709 (19)	C18—C19	1.205 (2)
C3—C4	1.438 (2)	C19—Si20	1.8366 (15)
C3—C18	1.426 (2)	Si20—C23	1.920 (3)
C4—C5	1.410 (2)	Si20—C230	1.834 (3)
C4—C9	1.402 (2)	Si20—C220	1.930 (3)
C5—C6	1.398 (2)	Si20—C22	1.804 (3)
C6—C7	1.387 (2)	Si20—C210	1.839 (4)
C6—H61	0.944	Si20—C21	1.856 (4)
C7—C8	1.406 (2)	C23—H231	0.930
C7—H71	0.960	C23—H232	0.930
C8—C9	1.382 (2)	C23—H233	0.930
C8—H81	0.959	C230—H2301	0.930
C9—H91	0.946	C230—H2302	0.930
C10—C11	1.518 (2)	C230—H2303	0.930
C10—H102	0.981	C220—H2201	0.930
C10—H101	0.969	C220—H2202	0.930
C11—H111	0.951	C220—H2203	0.930
C11—H112	0.965	C22—H221	0.930
C11—H113	0.971	C22—H222	0.930
C12—C13	1.3974 (19)	C22—H223	0.930
C12—C17	1.397 (2)	C210—H2101	0.930
C13—C14	1.382 (2)	C210—H2102	0.930
C13—H131	0.970	C210—H2103	0.930
C14—C15	1.390 (2)	C21—H211	0.930
C14—H141	0.953	C21—H212	0.930
C15—C16	1.385 (2)	C21—H213	0.930
			
C2—N1—C5	108.70 (11)	C16—C17—H171	119.9
C2—N1—C10	127.47 (11)	C3—C18—C19	178.42 (15)
C5—N1—C10	123.51 (11)	C18—C19—Si20	173.57 (14)
N1—C2—C3	109.21 (12)	C19—Si20—C23	106.48 (10)
N1—C2—C12	123.97 (12)	C19—Si20—C230	110.76 (12)
C3—C2—C12	126.81 (12)	C19—Si20—C220	108.16 (11)
C2—C3—C4	107.12 (12)	C23—Si20—C220	132.96 (15)
C2—C3—C18	125.82 (13)	C230—Si20—C220	108.21 (17)
C4—C3—C18	127.04 (13)	C19—Si20—C22	110.27 (12)
C3—C4—C5	106.57 (12)	C23—Si20—C22	109.80 (16)
C3—C4—C9	133.70 (14)	C230—Si20—C22	82.80 (18)
C5—C4—C9	119.73 (13)	C19—Si20—C210	108.84 (18)
C4—C5—N1	108.38 (12)	C230—Si20—C210	111.74 (18)
C4—C5—C6	121.89 (13)	C220—Si20—C210	109.0 (2)
N1—C5—C6	129.71 (13)	C22—Si20—C210	129.2 (2)
C5—C6—C7	117.15 (14)	C19—Si20—C21	108.08 (15)
C5—C6—H61	121.1	C23—Si20—C21	108.43 (18)
C7—C6—H61	121.8	C230—Si20—C21	128.55 (18)
C6—C7—C8	121.64 (14)	C220—Si20—C21	89.87 (18)
C6—C7—H71	119.2	C22—Si20—C21	113.51 (18)
C8—C7—H71	119.1	Si20—C23—H231	109.7
C7—C8—C9	120.98 (14)	Si20—C23—H232	108.8
C7—C8—H81	119.3	H231—C23—H232	109.5
C9—C8—H81	119.7	Si20—C23—H233	109.9
C4—C9—C8	118.56 (14)	H231—C23—H233	109.5
C4—C9—H91	120.4	H232—C23—H233	109.5
C8—C9—H91	121.0	Si20—C230—H2301	109.5
N1—C10—C11	112.70 (13)	Si20—C230—H2302	109.4
N1—C10—H102	107.6	H2301—C230—H2302	109.5
C11—C10—H102	109.2	Si20—C230—H2303	109.5
N1—C10—H101	108.2	H2301—C230—H2303	109.5
C11—C10—H101	109.2	H2302—C230—H2303	109.5
H102—C10—H101	110.0	Si20—C220—H2201	109.7
C10—C11—H111	108.8	Si20—C220—H2202	108.8
C10—C11—H112	110.5	H2201—C220—H2202	109.5
H111—C11—H112	109.4	Si20—C220—H2203	110.0
C10—C11—H113	109.3	H2201—C220—H2203	109.5
H111—C11—H113	108.2	H2202—C220—H2203	109.5
H112—C11—H113	110.6	Si20—C22—H221	109.0
C2—C12—C13	118.60 (13)	Si20—C22—H222	109.8
C2—C12—C17	122.61 (12)	H221—C22—H222	109.5
C13—C12—C17	118.64 (13)	Si20—C22—H223	109.6
C12—C13—C14	120.46 (15)	H221—C22—H223	109.5
C12—C13—H131	118.7	H222—C22—H223	109.5
C14—C13—H131	120.8	Si20—C210—H2101	109.6
C13—C14—C15	120.59 (14)	Si20—C210—H2102	109.4
C13—C14—H141	120.5	H2101—C210—H2102	109.5
C15—C14—H141	118.9	Si20—C210—H2103	109.4
C14—C15—C16	119.49 (14)	H2101—C210—H2103	109.5
C14—C15—H151	118.8	H2102—C210—H2103	109.5
C16—C15—H151	121.7	Si20—C21—H211	108.7
C15—C16—C17	120.15 (14)	Si20—C21—H212	109.7
C15—C16—H161	120.2	H211—C21—H212	109.5
C17—C16—H161	119.7	Si20—C21—H213	110.0
C12—C17—C16	120.67 (13)	H211—C21—H213	109.5
C12—C17—H171	119.5	H212—C21—H213	109.5

### Hydrogen-bond geometry (Å, º)

Cg4 and Cg5 are the centroids of the pyrrole and benzene rings, respectively, of indole ring system.

**Table:**

*D*—H···*A*	*D*—H	H···*A*	*D*···*A*	*D*—H···*A*
C10—H102···*Cg*5^i^	0.98	2.66	3.3512 (18)	128
C16—H161···*Cg*5^ii^	0.95	2.79	3.5122 (17)	133
C17—H171···*Cg*4^ii^	0.95	2.81	3.4515 (17)	125

Symmetry codes: (i) −*x*+5/2, *y*+3/2, −*z*+3/2; (ii) −*x*+5/2, *y*+5/2, −*z*+3/2.

## References

[bb1] Betteridge, P. W., Carruthers, J. R., Cooper, R. I., Prout, K. & Watkin, D. J. (2003). *J. Appl. Cryst.* **36**, 1487.

[bb2] Bruker (2001). *SADABS* Bruker AXS Inc. Madison, Wisconsin, USA.

[bb3] Bruker (2006). *APEX2* and *SAINT* Bruker AXS Inc. Madison, Wisconsin, USA.

[bb4] Huang, X.-H., Zhang, Q.-F. & Sung, H. H. Y. (2004). *Acta Cryst.* E**60**, o488–o489.

[bb5] Hussain, M., Tengho Toguem, S.-M., Ahmad, R., Tùng, Đ. T., Knepper, I., Villinger, A. & Langer, P. (2011). *Tetrahedron*, **67**, 5304–5318.

[bb6] Prateeptongkum, S., Driller, K. M., Jackstell, R., Spannenberg, A. & Beller, M. (2010). *Chem. Eur. J.* **16**, 9606–9615. 10.1002/chem.201000369 20521287

[bb7] Rives, A., Baglai, I., Malytskyi, V., Maraval, V., Saffon-Merceron, N., Voitenko, Z. & Chauvin, R. (2012). *Chem. Commun.* **48**, 8763–8765. 10.1039/c2cc34176j 22836347

[bb8] Ruiz, M., Sánchez, J. D., López-Alvarado, P. & Menéndez, J. C. (2012). *Tetrahedron*, **68**, 705–710.

[bb9] Seferoğlu, Z., Hökelek, T., Şahin, E. & Ertan, N. (2007*a*). *Acta Cryst.* E**63**, o148–o150.

[bb10] Seferoğlu, Z., Hökelek, T., Şahin, E. & Ertan, N. (2007*b*). *Acta Cryst.* E**63**, o568–o570.

[bb11] Sheldrick, G. M. (2008). *Acta Cryst.* A**64**, 112–122. 10.1107/S0108767307043930 18156677

[bb12] Shiri, M. (2012). *Chem. Re*v. **112**, 3508–3549. 10.1021/cr2003954 22390139

[bb13] Watkin, D. J., Prout, C. K. & Pearce, L. J. (1996). *CAMERON.* Chemical Crystallography Laboratory, Oxford, England.

